# Effects of Breastfeeding on Maternal Body Composition in Moroccan Lactating Women during Twelve Months after Birth Using Stable Isotopic Dilution Technique

**DOI:** 10.3390/nu13010146

**Published:** 2021-01-04

**Authors:** Baha Rabi, Kaoutar Benjeddou, Mohamed Idrissi, Anass Rami, Bouchera Mekkaoui, Asmaa El Hamdouchi, Hasnae Benkirane, Amina Barkat, Naima Saeid, Khalid El Kari, Hassan Aguenaou

**Affiliations:** 1 Joint Research Unit in Nutrition and Food, URAC 39 (Ibn Tofail University-CNESTEN) Regional Designated Center of Nutrition (AFRA/IAEA), Rabat-Kenitra 14000, Morocco; rabibaha@gmail.com (B.R.); idrissom@gmail.com (M.I.); anassrami13@gmail.com (A.R.); bouchramekkaoui40@gmail.com (B.M.); asmaaelhamdouchi@gmail.com (A.E.H.); benkirane.hasnae@uit.ac.ma (H.B.); saeid_na@yahoo.com (N.S.); khalidelkari@yahoo.fr (K.E.K.); AGUENAOU.Hassan@uit.ac.ma (H.A.); 2 Ibn Sina University Hospital Center, Children’s Hospital Rabat, Rabat 10000, Morocco; barakatamina@hotmail.fr

**Keywords:** breastfeeding, weight loss, deuterium oxide, postpartum, human milk

## Abstract

Background: Exclusive breastfeeding during the first six months of an infant’s life is an important factor for their optimal growth and health. Breastfeeding also has maternal benefits and can assist with postpartum weight loss. As shown by previous studies, postpartum weight retention can contribute to obesity. Objective: To quantify the human milk and evaluate the effect of breastfeeding on maternal weight loss during the 12 months postpartum. Method: This study included 70-mother–baby pairs. Infants’ intake of human milk and water from other sources, as well as the body composition of the mothers, were measured at the 1st, 3rd, 6th, 9th and 12th month postpartum by using the deuterium oxide dose-to-mother technique. Results: There was a significant change in the mothers’ body composition between the first and twelfth months in exclusive breastfeeding women compared to not-exclusive ones. Similarly, the difference between the quantities of human milk intake was highly significant in exclusive breastfeeding women compared to women who were not exclusively breastfeeding. Conclusion: Our results showed that exclusive breastfeeding for twelve months has a significant effect on postpartum weight loss among Moroccan women and that it is an effective way to control overweight and obesity among lactating women.

## 1. Introduction

Healthy eating practice plays an important role in the growth of an infant and helps ensure good development and better health during the first years of life. Therefore, breastfeeding is important for infant feeding during the first months of life. The World Health Organization (WHO) recommends exclusive breastfeeding (EB) for the first six months of the infant’s life and it is recommended to be maintained until the age of two or even beyond according to the willingness of the mother [1].

Numerous studies worldwide have confirmed the superiority of breast milk and its various health benefits, for both, the mother and the child [2]. Pregnancy is a factor in increasing obesity for women [3]. Up to 20% of women retain five kilogram or more of the weight they acquired during pregnancy [4]. Furthermore, already overweight or obese women have a high risk of keeping the gained weight after childbirth [5,6] contributing to the increase in the incidence of overweight and obesity worldwide, associated to an important increased risk to develop diabetes, heart diseases, metabolic disorders and cancer [7,8]. Therefore, this underscores the reason for evaluation of postpartum changes in the mother’s body composition, as it provides important information about the mother’s nutritional status [9,10]. Ultimately, the monitoring of weight gain which eventually leads to obesity becomes a priority in public health and nutrition policy.

Overweight and obesity are usually determined by the body mass index (BMI). But several studies confirmed its limitations to determine objectively the extent of obesity in societies [11]. In addition, it is still a controversy about the ideal indicator to measure overweight or obesity; as many scientists recommend fat mass percentage (FM%) against other authors who have raised its inefficiency as an indicator to determine overweight and obesity, because of its association to the fat free mass compartment variation and does not take account of individual’s size [12,13,14,15]. However, fat mass index (FMI) seems to be more interesting among the community of scientists and to be more relevant to assess excess of fat and its associations to illness [11,15,16].

In Morocco, the last survey on population and family health conducted in 2018 revealed that EB in children under six months is an area that has seen significant improvement in the past seven years: 35% of children against only 27.8% in 2011 according to mothers’ reports [17,18]. The main reason to not exclusively breastfeed until the age of six months is mainly due to changes in the family structure, in particular the lack of psychological support from the family circle, the occurrence of a new pregnancy, mother’s health, returning to work, and aesthetic concerns [19], in addition to the development of the dairy product industry, its aggressive marketing and insufficient training on breastfeeding practices provided by health professionals [20].

The “dose-to-mother” deuterium oxide dilution technique is an isotope dilution technique, accurate and non-invasive, to assess, at the same time, the pattern of breastfeeding (exclusive or not exclusive), the quantity of human milk (HM) consumed by breastfed infants, the quantity of water from sources other than human milk, as well as the body composition of the mother [21,22,23]. To quantify the HM, the method measures the turnover rate of body water based on the transfer of deuterium from mother to the child through the HM, after an oral dose of deuterium oxide (stable isotope) provided to the lactating mother. The method does not involve any HM sampling associated with the usual life style of the mother-infant pair. For body composition, the technique allows the evaluation of the mother’s total body water, from which her body composition can be calculated using a two-compartment pattern [21,22,23]. The relationship between breastfeeding and postpartum body weight loss or retention is inconclusive for many reasons. The main reasons for this discrepancy could be attributed to differences in study protocols (duration of breastfeeding, pattern of breastfeeding, etc.), methods used to measure different body compartments (anthropometry, skinfolds, isotope dilution, etc.), adjustment for some potential confounding factors (energy expenditure, marital status, smoking, education, etc.) and collecting data (direct collection or self-reporting) [24].

Thus, the objectives of this study were: (1) to quantify the intake of human milk and water from other sources than human milk by breastfed infants; (2) to assess the body changes using different obesity indicators in lactating mothers over a period of one year after childbirth; and (3) to determine the effect of the type of breastfeeding on the prevention of obesity.

## 2. Materials and Methods

### 2.1. Study Design and Subject

This is a descriptive longitudinal study in which mothers and their children are followed for one year after birth at the Health Center Lalla Meryem, Casablanca, Morocco, between 2012 and 2015. The mothers were aged 19–45, had a single pregnancy and were in good health without complications from pregnancy. Their babies were born after a full term, in good health and not suffering from any form of malnutrition. All subjects gave their informed consent for inclusion before they participated in the study. The study was conducted in accordance with the Declaration of Helsinki, and the protocol was approved by the Ethics Committee of the Moroccan Ministry of Health under the number N° 1487 23/12.

### 2.2. Socioeconomic Assessment

A questionnaire was used to gather socioeconomic data relevant to the families. Questionnaires were addressed to the mothers at the beginning of the study. They included information on parental education, household size, etc.

### 2.3. Sample Size

Calculation of the sample size required an isotopic breastfeeding evaluation study, with a known standard deviation of breastfeeding from breastfed babies and a defined difference between the two groups i.e., exclusively breastfed and not exclusively breastfed. As this is the first study of its kind in Morocco and in the Arab world, no information concerning the quantification of breast milk is available. In this regard, a standard deviation (σ) equal to 2043 g/day of breast milk and a difference (δ) between the groups equal 203 g of breast milk were used based on the results of Medoua et al., 2012 and Haisma et al., 2003 respectively [23,25]. For a power of 80% and a significant level of 0.05, with two study groups, the required sample size (n) can be calculated using the following equation (formula N° 1):

N = 2 × f × (σ/δ)^2^ (1)

where f (= 7.85) is the multiplication factor for a power of 80% and an α = 0.05. So:

N = 2 × 7.85 × (204.3/203)^2^ = 16 (2)

To achieve statistically significant results at the end of study, we would need a total of 32 mother-infant pairs. To account for a drop-out rate of ten percent, we needed 36 mother-infant pairs.

### 2.4. Anthropometric Measurements

The mothers’ weight was measured with an accuracy of 0.1 kg using an electronic balance (SECA 813). The mothers were invited to step on the scale with minimum of clothing possible. Height was measured only at the start of the study with a vertical measuring rod of 0.1 cm precision (SECA 203). The body mass index (BMI) of mothers was calculated according to the formula; (body mass (kg)/body height (m^2^)). The weight of babies was determined using the electronic baby scale (SECA 383) with a precision of 0.001 kg and they were weighed without clothing. The height of the babies was measured using an infant meter (SECA 417) with a precision of 0.1 cm. The infants’ Z-scores were calculated using the WHO Anthro Survey Analyzer [26].

### 2.5. Principle, Preparation and Administration of Deuterium Oxide Doses

The “dose-to-mother” technique was used to assess breast milk practices and intake [21]. The amount of breast milk consumed or the human milk intake (HMI) by the baby for 14 days was assessed using the isotopic dilution technique with deuterium oxide (^2^H_2_O). Deuterium (^2^H) is a stable isotope of hydrogen and it is not radioactive. It is administered orally to the mother as deuterium oxide. Deuterium oxide is metabolized in the body similarly to water and is dispersed as body water within few hours. The latter can be taken from saliva, urine, plasma or breast milk. Deuterium enrichment is measured by Fourier transform infrared spectroscopy (FTIR).

The doses were prepared per batch according to the needs. The 30 g doses of deuterium oxide (99.9% pure ^2^H_2_O, Aldrich, Casablanca, Morocco) were prepared in clean, dry 60 mL polypropylene (Nalgene™) bottles. Before administering a dose of deuterium oxide, it was ensured that the “pre-dose” saliva samples from the mother and baby were taken to determine the natural abundance of the deuterium.

The solutions of deuterium oxide were mixed by successive inversion of the bottles in order to avoid any condensation inside the lid. The dose was immediately consumed.

The dose was only taken by the mother using an oral straw. Once the bottle had been emptied, about 50 mL of drinking water was added to the bottle, to mix any remaining deuterium in the bottle and the mother consumed the water. The operation was repeated twice in order to remove all the deuterium contained in the bottle. After administration, saliva samples (at least two mL) were collected from both the mother (stirred the cotton ball in the mouth for two minutes until soaked) and the baby (the saliva is collected using a cotton swab rolled up with a piece of cotton; we put the cotton in the baby’s mouth until the cotton is soaked. Saliva samples were collected at time t = 0, before the administration of the dose of deuterium, and at one, two, three, four, thirteen and fourteen days after administration of the deuterium oxide dose, the date and time were marked on the monitoring sheet and on the bottle, which was be kept until the saliva samples were analyzed. The samples were placed in a cooler kept at around 4 °C and transported to the laboratory. They were then aliquoted and stored in the freezer at −86 °C until further analysis.

The deuterium concentration of the saliva samples was measured by infrared Fourier transform spectrometry (FTIR, Agilent model 4500s).

### 2.6. Classification of Breastfeeding

There were two categories of breastfeeding patterns according to the quantity of non-milk oral intake or non-breast milk water intake or water from other sources than human breast milk (NMOI) which is the quantity of water from other sources than breast milk; indeed, the EB is defined as NMOI < 86.6 g/day and not-exclusive breastfeeding (NEB) is defined as NMOI ≥ 86.6 g/day [27,28].

### 2.7. Body Composition of Mothers

Women’s total body water (TBW, kg) was calculated from saliva enrichment, free body fat (FFM, kg) was determined from TBW using a non-aqueous exchange factor 1.041, and body fat (FM, kg) was calculated as the difference between the mother’s body weight (kg) and FFM (kg) [20]. According to the previous study, the overweight and obesity among women were defined as FM% > 30% and > 35% respectively [29,30,31]. The FM index (FMI) have a similar concept to the BMI: FMI = FM (kg)/height (m^2^). The excess of fat among women was defined as FMI > 7.93 kg/m^2^ [32].

### 2.8. Statistical Analysis

The data were entered on Microsoft Office Excel version 2017 and analyzed using IBM SPSS software version 20.0. For data quality control and to evaluate the square root of the mean squared error, (MSE) was used to evaluate the modeled data fit which represents the variation between the measured and model-predicted enrichments of deuterium in the mother and baby. The MSE has to be less than 60 mg/kg [33].

Means were compared using Student test and proportions were compared using the Fisher test. The difference was considered significant for a value of *p* ≤ 0.05.

## 3. Results

### 3.1. Subjects

**Figure 1:**
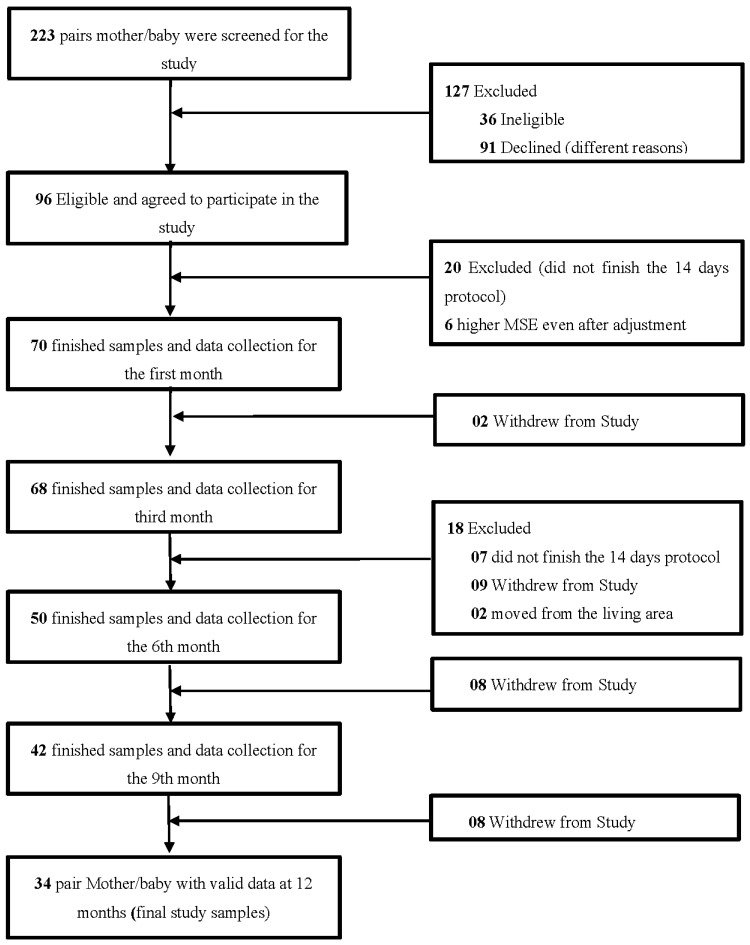
Study recruitment design. For different reasons 62 couples were excluded after their enrolment in the study from which only 27 withdrew.

The study started with 96 pairs of mothers/babies of which 34 completed the study. The main reason of the dropout was the sampling time over 14 days. There were nine pairs who dropped out due to personal reasons, while two pairs moved from their living area (Figure 1).

### 3.2. Anthropometric Characteristics of Infants during the Study

The anthropometric characteristics of the infants over the course of the study showed that the babies were growing normally. The average weight varied from 4.7 to 9.5 kg between the first and the twelfth month. In addition, babies’ average stature varied from 52.6 to 73.6 cm from the first and the twelfth month (Table 1).

### 3.3. Breast Milk and Other Fluids Intake

The average of HMI increased between the first and the sixth month. During the first month postpartum HMI was equal to 437.8 ± 110.7 g/day. At the end of the third month, it was around 585.6 ± 146 g/day, while in the sixth month it was equal to 604.2± 142.5 g/day (*p* = 0.07). The average milk intake gradually decreased to 435.3 ± 100.7 g/day in the twelfth month of the study (*p* < 0.0001). There was a gradual increase in the NMOI according to the age of the baby. During the first month, the quantity of NMOI was equal to 142.0 g/day and it reached 659.8 g/day at twelfth month. Thus, the total quantity of water consumed (TWI) daily by babies was 563.3, 751.4, 846.4, 935.0 and 1078.3 g during the first, the third, the sixth, the ninth and the twelfth months respectively (Table 2).

**Figure 2:**
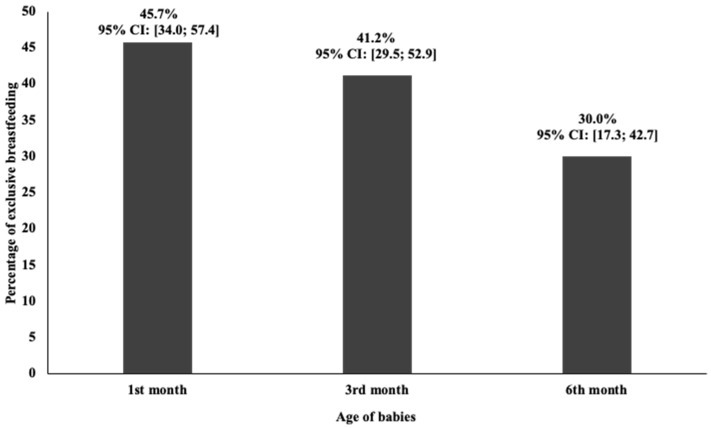
Rate of exclusive breastfeeding at 1st, 3rd and 6th months. Results presented as percentage with its confidence interval at 95% (95% CI).

Based on the quantity of NMOI, the rate of EB under six months was 45.7%, 41.2% and 30% during the first, the third and sixth month respectively (Figure 2).

Table 3 shows the average intake of breast milk, NMOI and TWI by breastfeeding pattern during the first, the third and the sixth month of the babies’ life. The intake of human milk was 492.4 ± 109.1, 688.2 ± 94.3 and 733.3 ± 88.8 g/day respectively in exclusively breastfed children. In the case of not exclusively breastfed babies, the quantity of milk was 383.7 ± 112.2, 531.3 ± 132.7 and 548.9 ± 123.7 g/day respectively. The differences were highly significant between the different patterns of breastfeeding (*p* < 0.003) during the first six months of the babies’ life. The NMOI was more than 8.0, 8.8 and 11.8 times higher among the not exclusively than exclusively breastfed children respectively during the first, the third and the sixth month; the difference is statistically significant (*p* < 0.001) (Table 3). On the other hand, consumption of total water was statistically higher among the not exclusively breastfed group during the six first months of babies’ life (*p* ≤ 0.002) except during the sixth month, when the difference is statistically not significant (*p* = 0.08) with a higher trend in not exclusive breastfeed children (Table 3).

### 3.4. Assessment of the Body Composition of Mothers

**Figure 3:**
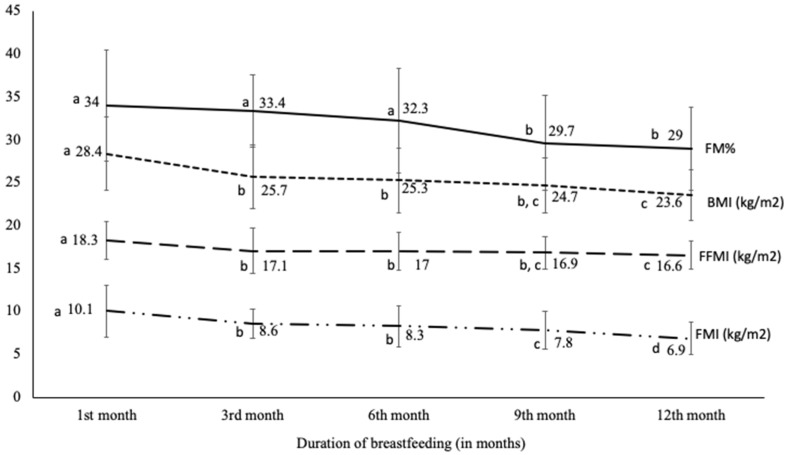
Longitudinal changes in: mothers’ body mass index, mothers’ fat mass percentage, mothers’ fat mass index and mothers’ fat free mass index from 1 to 12 months after birth measured with deuterium dilution technique. BMI: body Mass Index in kg/m^2^; FM%: fat mass percentage, FMI: fat mass index in kg/m^2^; FFMI: fat free mass index in kg/m^2^; a, b, c and d: for each variable separately, the values with the same letters are statistically not different however, the values with different letters are statistically different.

The variation in the BMI, the FM%, the FMI and the FFMI of the mothers is presented in Figure 3. The average of BMI decreased from 28.4 kg/m^2^ in the first month to 23.6 kg/m^2^ a year later (*p* < 0.001). The most important decrease was observed during the first six months. No significant change was recorded between the sixth and the ninth months. Moreover, during the period of the study, the average of BMI remained in the over-weight category practically until the ninth month. According to FM%, it decreased from 34% in the first month to 29% after twelve months (*p* < 0.001). No significant change was observed during the six first month. At the ninth month, the average of FM% decreased significantly from the overweight class to a normal class (29.7%). Their state remained stable until the twelfth month. No significant difference was observed between the ninth and the twelfth months (Figure 3). To obtain more relevant results, we used the FMI which indicated a non-excess fat around the ninth month with a highly significant change between the first (10.1 kg/m^2^) and the twelfth months (6.9 kg/m^2^) (*p* < 0.001). An important decrease was also observed during the first three months after delivery. FFMI, which indicates the variation of the lean body mass, shows an important decrease between the first (18.3 kg/m^2^) and the third month (17.1 kg/m^2^) which is similar to the others indicators (BMI and FMI). The values of FFMI were constant between the third and the ninth months then decreased significantly at the twelfth month in comparison to the sixth month (Figure 3).

**Figure 4:**
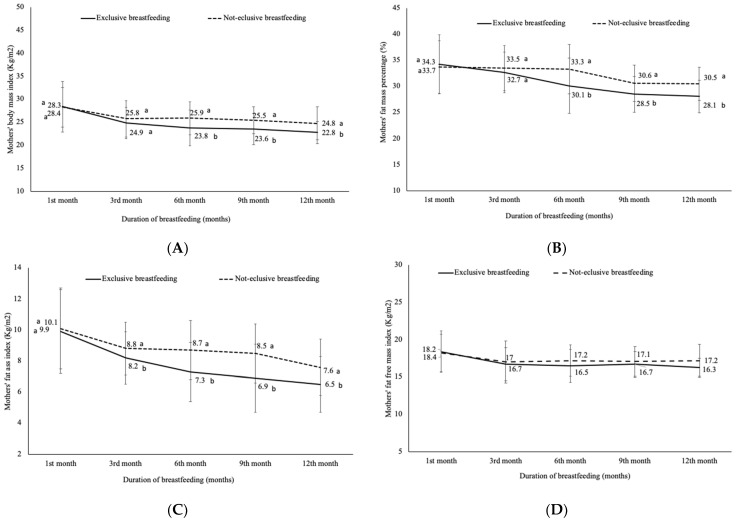
Longitudinal changes in: (**A**) mothers’ body mass index, (**B**) mothers’ fat mass percentage, (**C**) mothers’ fat mass index and (**D**) mothers’ fat free mass index during one year after delivery according to the breastfeeding pattern. BMI: body mass index in kg/m^2^; FM%: fat mass percentage; FMI: fat mass index in kg/m^2^; FFMI: fat free mass index kg/m^2^; a, b: represent the significance level (values with the same letters are statistically not different however, the values with different letters are statistically different) of the comparison at each point time.

The analysis of different indicators of excess of fat mass according to the breastfeeding pattern during twelve months indicates that the BMI decreased from the overweight class (28 kg/m^2^) to a normal class (24.9 kg/m^2^) during the first three months in the exclusively breastfed group, unlike the not exclusive breastfed group whose BMI returned to normal class at the end of the year (24.8 kg/m^2^). The difference was statistically not significant during the first three months between groups (*p* > 0.1) (Figure 4A). Within the exclusively breastfed group the BMI decreased significantly (*p* = 0.025) between the third (24.9 kg/m^2^) and the twelfth months (22.8 kg/m^2^), unlike the not exclusively breastfed group where the BMI remained unchanged (Figure 4A).

The FM% showed an important decrease between the first (34.3%) and the sixth (30.1%) month in the exclusively breastfed group and reached a normal category before the ninth month (Figure 4B). In not exclusively breastfed group, the average of FM% was constant during the six first months (FM% > 33.3%) and decreased significantly at the ninth month (30.6%) but remained in the overweight category until the end of the year (Figure 4B). The differences between groups are statistically significant starting from the sixth month.

Regarding FMI, it seems that the average of FMI reached a normal value after the third month in the exclusively breastfed group and decreased significantly during the year to reach a value of 6.5 kg/m^2^ at the twelfth month. No difference between the ninth and the twelfth months was observed. In the case of the not exclusively breastfed group, the FMI decreased significantly during the first three months and reached the normal class at the end of the year (7.6 kg/m^2^). No difference has been observed between the third and the ninth months (Figure 4C). For the FFMI, it seems that it was not influenced by the breastfeeding pattern because no difference was recorded between the two breastfeeding groups (Figure 4D).

## 4. Discussion

In our study, we followed the breastfeeding practices of 34 mother/baby pairs for twelve months postpartum. The use of the deuterium oxide dose technique to the mothers makes it possible to determine the mothers’ body composition, to quantify the intake of human milk, and to determine the pattern of breastfeeding [23,34].

Furthermore, we reported an objective estimation of HMI by using isotope technique during twelve months of life at five babies ages one, three, six, nine and twelve months. For many years, the ability to quantify HMI has been a challenge for researchers particularly to obtain good quality data. This could be due to the assessment methods employed especially the weighing method and the acceptable limit of the estimated error of the model in the case of the isotopic technique. In 2014, the IAEA (International Atomic Energy Agengy) standardized the estimation error of the calculation model by fixing the acceptable limit of MSE to 60 mg/kg which was adhered in our study.

During twelve months, the quantity of HM from our data is lower than reported by previous studies in developed countries for babies aged three to six months [35] and even in comparison with the pooled data from the Da Costa study in 2010 using the isotope technique [36]. However, they are in agreement with those previously reported for developing countries by WHO for EB or no EB infants except for babies aged one and twelve months when the quantity of HM in our data is lower [37].

When exclusively breastfed, a baby usually consumes 750 to 800 mL of milk every day at the age of six months. From nine to twelve months, the baby can still take about 500 mL per day, which provides about half of the daily calories [38]. At six months, the amount of breast milk consumed by exclusively breastfed infants is very variable. The amount consumed by breastfed infants exclusively from this study (733.3 ± 88.8 g/day), is similar to that reported in Pakistan (757 ± 249 g/day) [39] or in USA by Stuff in 1986 (764 ± 88 g/day) [40] and Pao in 1980 (737 g) [41] and is lower than that reported in Mexico (869 ± 150 g/day) [42].

After six months, breast volume, milk production and storage capacity decrease [38] which was also confirmed by our study, thus, the introduction of other foods (food diversification) which perhaps explains the decrease in the quantity HM consumed at nine and twelve months and the increase of NMOI and the TWI.

Regarding the EB rate at six months (30%) in our studied group, it was under the expectation of the national program for promoting breastfeeding, which is 50% by 2025. At the national level the rate of EB based on mother reporting is 35% [18]. This was in line with the knowledge that the validity of the mother reporting method to assess the rate of EB has been questioned over time following its overestimation due to self-reporting bias [43,44].

Direct markers of the nutritional status of nursing mothers, such as body composition, can provide direction for public health and nutrition policy at a national and multisectoral level. To our knowledge, information on changes in the body composition of breastfeeding women are limited and poorly documented especially in Arab and African countries. Pregnancy and postnatal period is one of the vulnerable periods in women’s life as they gain weight and it impacts their body composition [3]. The estimation of weight retention during the postpartum period range from 0.5 to 3.0 kg; whereas 14% to 20% of women gain five kg after six to 18 months postpartum than before their pregnancy [6,45]. In our study, we have used BMI, FM% and FMI to assess fat excess. It is well documented that BMI underestimate the excess of fat and has an important limit associated to its inability to distinguish between lean and fat body mass [46,47]. The FM% also showed an important limit particularly in the case of monitoring the change of the fat mass proportion, which is due to its direct association to the fat free mass compartment. However, FMI seems to be a more realistic index to assess and monitor excess of fat because of its independence from the fat free mass compartment and takes in a count the height [15].

In our study, it seems that all indicators of excess of fat (BMI, FM% and FMI) return to their normal categories around the ninth month after childbirth. The most important loss was observed during the first three months of babies’ life except for the FM% which decreased significantly around the ninth month. Another finding from our study is that breastfeeding impacted more on the FM compartment than the FFM compartment after the third month of breastfeeding, which was an indication of the protective effect of breastfeeding against fat retention after childbirth. This finding confirmed the rapid weight loss found by previous publications [48]. However, the effect of breastfeeding on weight loss is very controversial in the literature. Several studies have shown or suggested its positive impact on weight loss and protective effect against obesity, while other studies have shown no impact. Discrepancies originate from several factors which could be the integration of studies as well study designs (the study sample size, stage of lactation, the method of measurement of the weight and body composition and the child breastfeeding status), the life style of mothers, structure of families, sleep quality, psychological health of the mothers, weight gain before giving birth, level of physical activity, nutritional status of mother, cultural practices and food availability [24,47,49].

Furthermore, it seems that the pattern of breastfeeding influenced the body composition and fat retention. Our results show that fat loss among EB mothers tended to be higher for a short time compared to the other breastfeeding patterns. The women practicing EB return to a normal FM compartment from the third month whereas it is after the ninth month for women not practicing EB. However, the breastfeeding pattern didn’t show any impact on the FFM compartment. This result could be in line with the conclusion from other studies, which have found important postpartum weight loss as a result of fat loss in exclusively breastfeeding mothers compared to other forms of breastfeeding [48,50,51,52]. Other studies have also shown that EB protects against maternal obesity in affluent and transitioning populations, but it could contribute to maternal exhaustion in undernourished populations [53,54]. Several researches have shown that breastfeeding has significant health benefits for mothers particularly to reduce the risk of mothers with gestational diabetes developing type 2 diabetes, to reduce the risk of ovarian and breast cancer and could help mothers to lose weight after baby’s birth [55]. The findings from this paper highlight the importance of exclusive breastfeeding as a practice that could help mothers to lose weight during breastfeeding periods and therefore reduce the rate of obesity, its complications and subsequent diseases. Thus, contributing significantly to the improvement of the clinical and the public health. Our study had limitations. First, from the 6th month the number of mother/baby pair in the EB group is equal to 15 instead of 16. The second important limitation is the absence of a control group (non-lactating mothers) to eliminate the effects of other factors that may impact weight change among lactating mothers further that breastfeeding. Furthermore, several studies that reported a direct association between breastfeeding and weight loss have adjusted for potential confounding factors particularly pre-pregnancy weight or BMI, parity, energy expenditure or physical activity, diet or food intake, sleep deprivation and smoking [24,56]. Unfortunately, data on those factors was not collected in our study which did not allow us to make adjustments. However, other studies did not adjust for any factors [57,58,59,60,61].

## 5. Conclusions

This study is the first to be conducted in North Africa and to provide new information on the body composition of lactating women and the amount of breast milk consumed by infants for a period less than twelve months. In our study, the quantity of human milk consumed by babies seems to be lower than reported in developed countries, but similar to reported in developing countries particularly for babies aged three, six and nine months. It seems that breastfeeding contributes significantly to weight loss after childbirth, particularly fat loss for mothers who practice EB more than mothers who practice other patterns of breastfeeding. Hence, the need to encourage the practice of EB is of paramount importance in preventing and controlling obesity and thereby reducing and preventing other diseases.

## Figures and Tables

**Table 1:** Characteristics of infants and mothers according to baby’s age.

	1st Month	3rd Month	6th Month	9th Month	12th Month
Babies’ weight (kg)	4.7 ± 0.5	6.4 ± 1.0	8.1 ± 1.1	8.8 ± 0.9	9.5 ± 1.0
Babies’ height (cm)	52.6 ± 3.1	60.6 ± 3.5	64.9 ± 3.6	68.8 ± 4.7	73.6 ± 3.4
WHZ	1.27 ± 1.13	0.60 ± 1.34	1.40 ± 1.26	1.35 ± 1.40	0.57 ± 0.77
WAZ	0.28 ± 0.94	0.37 ± 1.27	0.55 ± 1.22	0.39 ± 1.00	0.18 ± 0.95
HAZ	−0.65 ± 1.58	0.08 ± 1.47	−0.72 ± 1.38	−0.88 ± 1.24	−0.47 ± 1.05
BAZ	0.95 ± 0.77	0.47 ± 1.24	1.23 ± 1.27	1.21 ± 1.41	0.63 ± 0.84
Mothers’ age (years) median [Min; Max]	30 [18; 41]	-	-	-	-
Mothers’ weight (kg)	73.8 ± 11.8	66.6 ± 11.9	64.9 ± 11.6	64.0 ± 12.1	62.3 ± 12.1
Mothers’ height (m)	1.59 ± 0.07	-	-	-	-
Mother’s education level		-	-	-	-
No formal school, or primary school	48.6%				
Secondary school	35.7%				
High school or university	15.7%				

All anthropometrics data are represented as means± SD; the age of mothers is represented as median (Min; Max) and the education level of mothers is the percentage of the distribution of different education level. WHZ: weight-for-height Z-scores; WAZ: weight-for-age Z-scores; HAZ: height-for-age Z-scores; BAZ: body-mass-index-for-age Z-scores. (*n* = 32, *n* = 28, *n* = 15, *n* = 15 and *n* = 15 at 1, 3, 6, 9 and 12 months respectively for the exclusive breastfeeding (EB) group) (*n* = 38, *n* = 40, *n* = 35, *n* = 27 and *n* = 19 at 1, 3, 6, 9 and 12 months respectively for the not-exclusive breastfeeding (NEB) group).

**Table 2:** Average intake (g/day) of human breast milk, water from sources other than breast milk and total water intake during the study period.

	HMI (g/Day)	NMOI (g/Day)	TWI (g/Day)	MSE (mg/kg)
**1st month**	437.8 ± 110.7	142.0 ± 145.5	563.3 ± 123.1	38,5 ± 14.5
**3rd month**	585.6 ± 146.0	188.3 ± 169.7	751.4 ± 106.1	32.2 ± 13.3
**6th month**	604.2± 142.5	265.4 ± 196.8	846.4 ± 107.7	28.8 ± 12.5
**9th month**	512.2 ± 141.3	442.5 ± 136.0	935.0 ± 119.2	39.9 ± 12.9
**12th month**	435.3 ± 100.7	659.8 ± 154.9	1078.3 ± 99.3	26.7 ± 11.4

Results are presented as means± SD. In 2014, the IAEA (International Atomic Energy Agency) standardized the estimation error (MSE) of the calculation model by fixing the acceptable limit of the MSE to 60 mg/kg. HMI: human milk intake; NMOI: water from sources other than breast milk; TWI: total water intake; MSE: mean squared error.

**Table 3:** Average intake (g/day) of breast milk and water from sources other than breast milk during the 1st, 3rd and the 6th month of the study according to exclusive and not exclusive breastfeeding.

	Exclusive Breastfeeding	Not-Exclusive Breastfeeding	*p*-Value
**1st month**			
HMI (g/day)	492.4 ± 109.1	383.7 ± 112.2	0.003
NMOI (g/day)	21.4 ± 21.8	252.6 ± 142.0	<0.001
TWI (g/day)	494.8 ± 108.1	621.5 ± 129.0	0.002
**3rd month**			
HMI (g/day)	688.2 ± 94.3	531.3 ± 132.7	<0.001
NMOI (g/day)	28.9 ± 25.4	255.5 ± 134.2	<0.001
TWI (g/day)	690.6 ± 94.3	765.9 ± 93.2	<0.001
**6th month**			
HMI (g/day)	733.3 ± 88.8	548.9 ± 123.7	0.003
NMOI (g/day)	45.0 ± 31.5	359.8 ± 157.4	<0.001
TWI (g/day)	750.1 ± 91.3	887.6 ± 84.9	0.08

Results are presented as means± SD. The results are presented only during the first six months of infant life because the exclusive breastfeeding is only practiced during this period. HMI: human milk intake; NMOI: water from sources other than breast milk; TWI: total water intake.
